# Antidiabetic Drugs in NAFLD: The Accomplishment of Two Goals at Once?

**DOI:** 10.3390/ph11040121

**Published:** 2018-11-08

**Authors:** Matteo Tacelli, Ciro Celsa, Bianca Magro, Aurora Giannetti, Grazia Pennisi, Federica Spatola, Salvatore Petta

**Affiliations:** Sezione di Gastroenterologia e Epatologia, DiBiMIS, University of Palermo, 90127 Palermo, Italy; matteo.tacelli@gmail.com (M.T.); celsaciro@gmail.com (C.C.); bianca_magro@hotmail.it (B.M.); auroragiannetti@gmail.com (A.G.); graziapennisi901@gmail.com (G.P.); federicaspatola1991@gmail.com (F.S.)

**Keywords:** non-alcoholic fatty liver disease, non-alcoholic steatohepatitis, Metformin, Thiazolidinediones, Liraglutide, hepatic cirrhosis

## Abstract

Non-Alcoholic Fatty Liver Disease (NAFLD) is the most common cause of chronic liver disease in Western countries, accounting for 20–30% of general population and reaching a prevalence of 55% in patients with type 2 diabetes mellitus (T2DM). Insulin resistance plays a key role in pathogenic mechanisms of NAFLD. Many drugs have been tested but no medications have yet been approved. Antidiabetic drugs could have a role in the progression reduction of the disease. The aim of this review is to summarize evidence on efficacy and safety of antidiabetic drugs in patients with NAFLD. Metformin, a biguanide, is the most frequently used drug in the treatment of T2DM. To date 15 randomized controlled trials (RCTs) and four meta-analysis on the use of metformin in NAFLD are available. No significant improvement in histological liver fibrosis was shown, but it can be useful in the treatment of co-factors of NAFLD, like body weight, transaminase or cholesterol levels, and HbA1c levels. A possible protective role in various types of cancer has been reported for Metformin. Thiazolidinediones modulate insulin sensitivity by the activation of PPAR-γ. The RCTs and the meta-analysis available about the role of these drugs in NAFLD show an improvement in ballooning, lobular inflammation, and perhaps fibrosis, but some side effects, in particular cardiovascular, were showed. GLP-1 analogues stimulate insulin secretion by pancreatic beta cell and inhibit glucagon release; Liraglutide is the most used drug in this class and significantly improves steatosis, hepatocyte ballooning and transaminase levels. Scanty data about the role of DPP-4 and SGLT inhibitors were published. No data about insulin effects on NAFLD are available but it was showed a possible association between insulin use and the development of solid neoplasms, in particular HCC. In conclusion, antidiabetic drugs seem to be promising drugs, because they are able to treat both NAFLD manifestations and diabetes, preventing worsening of hepatic damage, but data are still conflicting. All antidiabetic drugs can be safely used in patients with compensated cirrhosis, while insulin is the preferred drug in decompensated Child C cirrhosis.

## 1. Introduction

Non-Alcoholic Fatty Liver Disease (NAFLD), a spectrum of conditions ranging from simple steatosis to non-alcoholic steatohepatitis (NASH) and liver cirrhosis, is the most common cause of chronic liver disease in Western countries [1]. Prevalence of NAFLD is roughly 20–30% in the general population, but it reaches 55% in patients affected by type 2 diabetes mellitus (T2DM) [2,3]. Cohort studies suggest that presence of NAFLD at baseline is an independent predictor of the occurrence of T2DM [4,5]. At same time, presence of T2DM independently predicts the occurrence of fatty liver [6,7]. Insulin resistance plays a key role in pathogenic mechanisms of NAFLD and it acts as a trigger for progression of steatosis towards steatohepatitis, cirrhosis, and end-stage complications [2]. NAFLD is associated with higher risk of death, mainly due to cardiovascular disease (CVD), cancer and liver-related death and the risk increases according to fibrosis stage [8]. In spite of increasing epidemiological burden, to date no medication has been approved for treatment of NAFLD. Although healthy diet and habitual physical activity resulting in weight loss is advisable in all NAFLD patients, pharmacological treatment of progressive or active NASH remains an urgently unmet medical need. Treatment should aim to reduce liver-related mortality and progression towards cirrhosis and its complications [9]. Identification of adequate surrogate endpoints is essential to measure efficacy and effectiveness of pharmacological treatments of NASH, so regression or improvement of NASH histological lesions should be the goal to reach when new pharmaceutical approaches are tested.

**Figure 1:**
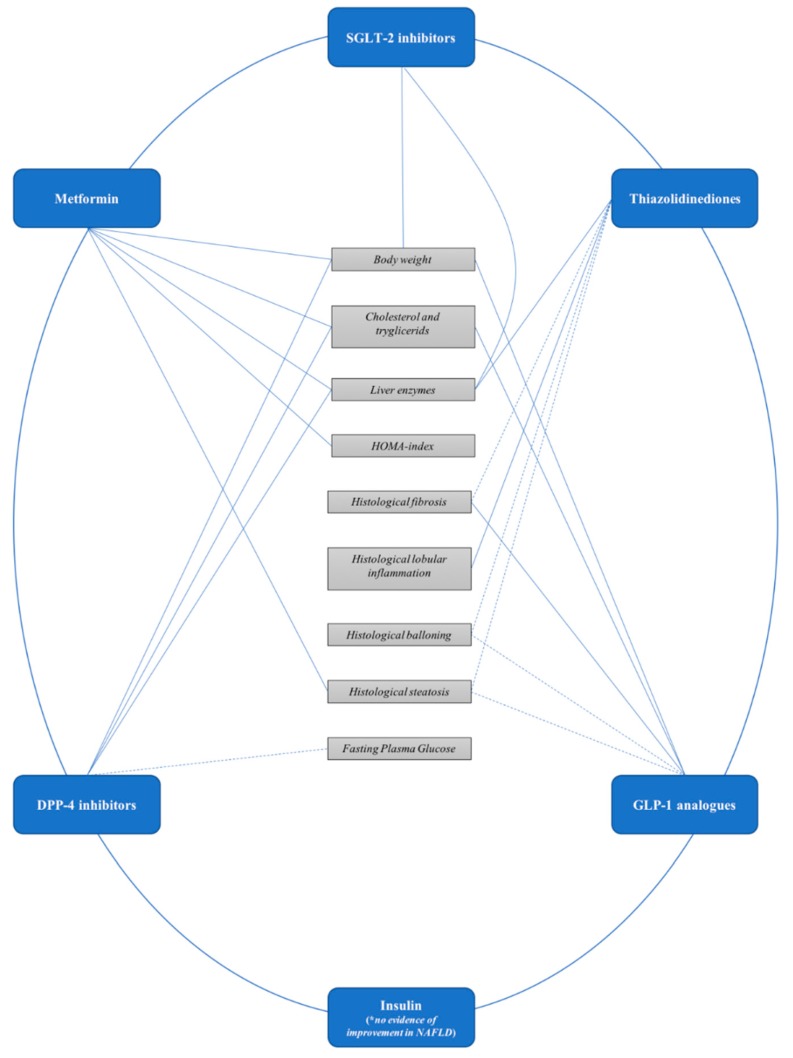
Effect of antidiabetic drugs on metabolic and liver outcomes in patients with non-alcoholic fatty liver disease. SGLT-2: sodium-glucose cotransporter 2; DPP-4: Dipeptidyl peptidase-4; GLP-1: Glucagon-like pepide-1.

The aim of this review is to summarize evidence on efficacy and safety of antidiabetic drugs in patients with NAFLD (Table 1 and Figure 1).

## 2. Insulin-Sensitizing Agents

Impaired response to insulin actions, or insulin resistance (IR) is the main pathogenic mechanism of NAFLD. IR acts on energy homeostasis increasing lipolysis, gluconeogenesis, and glycogenolysis, finally leading to high blood levels of free fatty acids (FFAs) and hyperglycaemia. Imbalance between synthesis and delivery of FFAs and the effects of hyperglycaemia on up-regulation of lipogenic transcription factors lead to accumulation of liver fat. Association between IR and endothelial dysfunction, systemic subclinical inflammation and oxidative stress contributes to increase the risk of CVD, also in non-diabetic patients [10]. Moreover, IR promotes the progression to NASH [11] and risk of liver and not liver cancer [11,12]. For this reasons, insulin-sensitizing drugs have been tested in diabetic and non-diabetic patients with NAFLD.

## 3. Metformin

Metformin is a biguanide glucose-lowering agent and represents the first-line choice for oral therapy in T2DM. This is due to its biological effects, consisting in decreasing: (1) hepatic gluconeogenesis, (2) intestinal glucose absorption, (3) total cholesterol/LDL (low-density lipoprotein)/triglycerides and (4) body weight; and in increasing: (1) glucose uptake in periphery, (2) muscle gluconeogenesis and (3) fatty acid oxidation. Its handling depends mainly on low rate of adverse events, represented especially by gastrointestinal effects (nausea and diarrhoea), worsening of renal function and lactic acidosis. 

It has been showed that metformin, reducing IR, has a positive impact on the development of metabolic syndrome and in reduction of CVD risk [13,14]. Features of randomized controlled trials evaluating metformin in NAFLD are reported in Table 1. To date 15 randomized controlled trials (RCTs) [15,16,17,18,19,20,21,22,23,24,25,26,27,28,29] on the use of metformin in NAFLD are available; in seven of them liver biopsies before and at the end of the study were obtained. To assess a systematic comparison between various studies, four meta-analysis [30,31,32,33] were performed. No significant improvement in liver histology was showed, in terms of liver fibrosis. However, metformin was showed to have a significant improvement in body weight, waist circumference, HOMA index, transaminase, FPG (fasting plasma glucose), blood cholesterol levels and HbA1c. These evidences suggest that role of metformin could be scanty in an overt NASH therapy, while it can be useful, in association with lifestyle intervention, in the preventive treatment of possible risk factors for NAFLD, maybe with a positive correlation between weight loss and improvements in hepatocellular injury and inflammation.

It is well-known that NAFLD and insulin resistance are associated with an increase in cancer development. But the use of metformin seems to have a protective role against all types of cancer, both hepatic [34,35] and non-hepatic ones (breast, colon, ovary, pancreas, lung and prostate) [36]. The molecular way of this risk reduction is still not clear, but it is thought to be due to inhibition of synthesis of reactive oxygen species (ROS) production as a consequence of its effects on mitochondrial function, and by regulating the AMP-activated kinase (AMPK)/mammalian Target of Rapamycin (mTORC1) pathway favoring the anti-proliferative effects of AMPK [37].

A possible role in the improvement of NAFLD/NASH has been attributed to the interaction between metformin and gut microbiome. In fact, metformin increases the production of butyrate[38] (a short chain fatty acid) from fiber-rich foods by the colonic bacteria. After binding to its receptor, butyrate activate AMPK way promoting lipolysis, fatty acid oxidation, glycogen synthesis, reduces glycolysis reduction and up-regulation of glucose transporter type 4 (GLUT4) [39,40]. Furthermore, metformin produces changes in gut microbiome that inhibit senescence mechanisms of the cells and, so, cancer development. For instance, the decreased amount of F. nucleatum could explain the lower incidence of colon cancer in metformin users [41].

## 4. Thiazolidinediones

Thiazolidinediones, such as pioglitazone and rosiglitazone, modulate insulin sensitivity by the activation of peroxisome proliferator-activated receptor (PPAR)-γ, that regulates the transcription of genes involved in lipid metabolism through the response of elements in promoter regions activated by ligands including fatty acids, eicosanoids, and oxidized forms of these molecules. The PPAR y receptor is most expressed in adipose tissue and lesser in the colon, kidney, liver, and small intestine in humans. Through different pathways, Glitazones modulate adipose tissue distribution, decreasing visceral fat, including hepatic fat, but they increase peripheral adiposity associated with weight gain that is a common side effect.

In steatohepatitis adipose tissue dysfunction, and insulin-resistance may play a pathogenetic role and for this reason glitazones could be used to treat this kind of patients. As reported in Table 1 Fifteen RCTs [19,22,26,42,43,44,45,46,47,48,49,50,51,52] studied their efficacy improving histological and clinical features of NASH. It is showed that Pioglitazone and Rosiglitazone, determinate a significant histologically improvement in steatosis and lobular inflammation, but no significant improvement was showed in terms of liver fibrosis, except for the RCT of 2006 [47] on Pioglitazone (where the fibrosis scores improved in the pioglitazone group—*p* = 0.002—, but the change from baseline did not differ significantly between the pioglitazone group and the placebo group—*p* = 0.08) and the recent trial of 2016 by Cusi et al. [52] in which pioglitazone (compared to placebo) was administered for 18 months with a dosage of 45 mg/daily. In this last case the diabetic population included in the study was 48%. Moreover, this is to date, the only trial where Thiazolidinediones (TZD) was administered for more than one year, so duration of treatment may play a role on histological findings in NASH.

Several meta-analyses [53,54,55,56] were performed to establish the possible role of TZD on histological findings in NASH patients and the results were almost contrasting. In fact, while all the meta-analyses agree with the benefic effect of TZD on lobular inflammation, the resolution of steatosis, fibrosis or ballooning is not clear. No significant improvement in liver fibrosis is showed, but in the subgroup of the trials where changes in lifestyle were added to TZD treatment there is a significant improvement. Furthermore, in two of 4 MA an improvement in steatosis and in ballooning was showed.

Principal side effects of TZDs are heart failure and peripheral edema. American Heart Association (AHA) and American Diabetes Association (ADA), suggest a careful evaluation of patients before starting treatment with TZD. In particular this type of therapy should be avoided in patients with HF symptoms and signs of New York Heart Association (NYHA) class III or IV. This is due to the evidence in various trials [57,58] that the risk of heart failure is increased in patients treated with TZDs, even if TZD-associated HF has not been linked with increased mortality [59]. This is confirmed by a meta-analysis of 2007 [60] that concluded that Rosiglitazone was associated with a significant increase in the risk of myocardial infarction and with an increase in the risk of death from cardiovascular causes. Subsequently, the FDA added a black box warning about myocardial ischemia to the drug’s label and it was voted by 13 of 33 members of the committee that Rosglitazone should be removed from the market. FDA has continued to monitor the drugs and found no new, relevant safety information. In 2013 FDA lifted restrictions on prescribing and dispensing rosiglitazone medicines after concluding that data did not show a higher risk of heart attack with rosiglitazone medicines compared with the standard type 2 diabetes drugs, metformin and sulfonylurea. The FDA has continued to monitor the drugs and found no new, relevant safety information and so it was concluded that the REMS (Risk Evaluation Mitigation Strategy) was no longer necessary [61].

As other important side effects, it was reported that patients undergoing TZD therapy have increased risk of developing bone fractures [62] in women (*p* < 0.001), but not in men (*p* = 0.83), and bladder cancer, especially in patients treated with pioglitazone respect to rosiglitazone [63]. On the other hand a meta-analysis showed that risk of cancer in patients treated with rosiglitazone was significantly lower than in placebo controls [64]. Furthermore many trials showed that TZDs treatment is associated with weight gain.

Finally, pioglitazone is the only agent of this class of drugs that can be used in patients with NAFLD, with a careful monitoring. In particular, it should be used ideally in patients with NASH and T2DM without heart failure or other contraindications to glitazones. 

## 5. Glucagon-Like Peptide-1 (GLP-1) Analogues

Glucagon-like pepide-1 (GLP-1) analogues are a class of drugs approved for the treatment of T2DM. GLP-1 is released from intestinal epithelial L-cells in response to meals and acts as agonist of GLP-1 receptor, stimulating insulin secretion by pancreatic beta cell, inhibiting glucagon release and maintaining glucose homeostasis. GLP-1 analogues are effective in lowering glucose blood levels, but they are showed to have also other pleiotropic extra-pancreatic effects, both at central and peripheral level. They decrease the appetite, delay gastric emptying, induces weight loss, improve cardiac function and have also hepatic effects [65]. In experimental animals treated with exenatide, it was reported a decrease in hepatic fat, probably mediated by improvement in lipid oxidation [66,67,68]. However, the presence of GLP-1 receptor in human hepatocytes is controversial and the mechanisms by which GLP-1 analogues act directly on liver, reducing hepatic steatosis, inflammation and fibrosis remain to be established [66,69].

Exenatide, a synthetic form of a hormone isolated from Gila monster saliva, was the first agent of this class to be approved for T2DM. Three RCTs conducted on diabetic patients [70] showed that exenatide significantly improves liver enzymes and reduces body weight. It was showed that exenatide in addition to insulin glargine compared to intensive insulin therapy is associated with significant improvement in body weight and liver enzymes [71]. However, its effect on histological outcomes was never tested.

Liraglutide is a long-acting GLP-1 analogue that can be administered once daily and it was recently licensed also for the treatment of obesity, both in US and in Europe. An individual patient data meta-analysis of six RCTs including more than 4000 patients with T2DM showed that twenty-six weeks of liraglutide treatment significantly improves liver enzymes and is safe and well tolerated [72]. The impact of liraglutide on hepatic histology was first assessed in LEAN trial, a multicentre phase II RCT that compared forty-eight weeks of subcutaneous liraglutide (1.8 mg/day) versus placebo in 52 patients with biopsy-proved NASH [73]. Primary endpoint was resolution of NASH (defined as disappearance of hepatocyte ballooning) without impairment of fibrosis, and was obtained in 39% of patients treated with liraglutide versus 9% of patients in placebo group (*p* = 0.019). No significant differences were observed in progression of fibrosis between two groups, although only 2 patients treated with liraglutide (versus 8 patients in placebo group) experienced worsening of hepatic fibrosis. Liraglutide significantly improved steatosis and hepatocyte ballooning, but no significant differences were seen in lobular inflammation and in NAFLD Activity Score. The benefit of liraglutide on histological outcomes is probably due to its direct hepatic effect and to weight loss and authors suggested a possible synergistic and multifactorial effect. Regardless of the severity of NASH, liraglutide showed a good safety profile, also in patients with cirrhosis. A sub-study of LEAN trial clarified some of metabolic effects of liraglutide: (1) reduction of free fatty acids concentration and peripheral lipolysis; (2) reduction of hepatic de novo lipogenesis; (3) reduction of hepatic glucose production and improvement of hepatic IR; (4) reduction of production of pro-inflammatory cytokines associated with hepatic fibrosis and increase of adiponectin levels [74]. When LEAN trial was designed, only 1.8 mg dose was available and 3 mg dose was subsequently approved for the treatment of obesity. More recently, a pilot randomized trial compared the 3 mg dose versus a structured weight-loss lifestyle intervention in obese Asian patients with NAFLD, showing a positive effect in improvement of body weight and liver enzymes in patients treated with liraglutide, but without significant differences in comparison with lifestyle intervention group [75]. However, it should be emphasized that these results have been obtained in a small cohort of patients with NAFLD and have to be confirmed in further large-scale studies.

## 6. Sodium-Glucose Cotransporter 2 (SGLT-2) Inhibitors

Sodium-glucose cotransporter 2 (SGLT-2) inhibitors reduce glucose reabsorption by kidney and also by bowel and heart. SGLT-2 is mainly present on the epithelial cells that line the S1 segment of the proximal contorted tubule and it is fundamental in promoting glycosuria. In this way, this mechanism of blood glucose level control is independent by secretion and insulin sensitivity, respect to other antidiabetic drugs.

Several pre-clinical studies conducted with animal experimental models showed that Ipragliflozin, Remogliflozin, Luseogliflozin, Canagliflozin, Empagliflozin, and Tofogliflozin [76,77,78,79,80,81,82,83,84] could be associated with hepatic steatosis improvement and, in some cases, also with liver fibrosis. In only one study [84] it was showed that Tofogliflozin could reduce the risk of progression to hepatocellular carcinoma. To date there are no studies regarding the role of SGLT-2 inhibitors in improvement of liver histology in NAFLD/NASH patients. Two RCTs [44,85] about the possible role of SGLT-2 inhibitors have been recently published.

In the study by Ito et al. 66 patients with type 2 diabetes and NAFLD were randomly assigned to receive ipragliflozin (*n* = 32) or pioglitazone (*n* = 34). While there were no differences in aminotransferase levels, HbA1c, and fasting plasma glucose, it was showed that only in the group of patients receiving ipragliflozin there was an improvement in visceral fat and body weight. Similarly in the study by Kuchay et al. it was showed that Empagliflozin could significantly reduce liver fat, measured by MRI-derived proton density fat fraction (MRI-PDFF), respect to control group (respectively *p* < 0.0001 vs. *p* = 0.057). It was also demonstrated an improvement in ALT levels, but not in gammaGT or AST.

One of the most important side effects in treatment with SGLT-2 inhibitors is the increased risk of developing urinary and genital infections. This can be explained by the sustained glycosuria that may facilitate the growth of pathogenic microorganisms. A meta-analysis [86] showed that gliflozins use is mildly associated with a 42% increase in genito-urinary infections (OR = 1.42, 95% CI: 1.06 to 1.90). It has also been reported a potential increased risk of developing malignancies, especially breast or bladder, but no studies have been confirmed this possibility [87]. Ketoacidosis, hypovolemia and increased cholesterol levels were reported as other possible minor side effects.

## 7. Dipeptidyl Peptidase-4 (DPP-4) Inhibitors

Dipeptidyl peptidase-4 is a membrane-associated peptidase, also known as CD26, capable to rapidly degrade incretins, deactivating them. Drugs blocking this enzyme are used in the treatment of diabetes mellitus, because they are able to prolong considerably biologic life of incretins; furthermore they increase the pool of active incretins and promote insulin production. The possible benefic role in NAFLD has been proposed in both in vitro and in vivo studies. In mouse models, Gemigliptin [88], Alogliptin [89], Teneligliptin [90], Sitagliptin [91], and Linagliptin [92], were able to alleviate hepatic steatosis, inflammation, hepatic lipogenesis, and insulin resistance. In light of these preliminary results the role of DPP-4 inhibitors in humans were tested and most of the studies were about sitagliptin. The first report was in 2012 and it was showed an improvement in glycosylated hemoglobin and in hepatic steatosis on MRI for a 67-year-old Asian woman with NAFLD treated with sitagliptin [93]. After that two RCTs [94,95] were published. In the first study 50 NAFLD patients were randomly assigned to receive sitagliptin 100 mg/day or placebo for 24 weeks. No significant differences for changes in alanine aminotransferase, aspartate aminotransferase, low-density lipoprotein, homeostatic model assessment insulin resistance, and MRE-derived liver stiffness were showed. The second RCT was published in 2017 and was aimed to test histologic and non-histologic changes in NAFLD patients treated with sitagliptin for 24 weeks. In comparison with placebo, sitagliptin did not show significant changes in biopsy fibrosis score, anthropometrics, liver enzymes, other adipocytokines, lipid profile, thrombosis parameters, or adipose distribution. Recently one more RCT [96] compared patients treated with sitagliptin and symbiotic versus patients treated with the only sitagliptin. The results of this trial are that sitagliptin-synbiotic produces greater improvement in FBS, AST, Cholesterol and LDL compared to sitagliptin alone in patients with NAFLD. Different results respect to sitagliptin were obtained in one RCT [97] of 2016 where 58 patients were randomly assigned to receive placebo or Vildagliptin 50 mg twice a day for twelve weeks; in this study significant improvement in BMI, triglycerides, cholesterol and aminotransferase levels was showed. DPP-4 inhibitors are almost well tolerated. The most common adverse reactions occurring in 5% of patients or more who received DPP-4 inhibitors are upper respiratory tract infection, nasopharyngitis, urinary tract infection, and headache. In patients treated with DPP-4 inhibitors in combination with other antidiabetics such sulfonylurea or insulin, risk of hypoglycemia is increased [98].

Finally, these data have been obtained in small cohorts and results remain weak and still not conclusive, needing further studies to substantiate the useful of DPP-4 inhibitors.

## 8. Meglitinides and Sulfonylureas

Sulfonylureas are largely used in the management of T2DM [99] and are currently positioned as second-line after failure of metformin monotherapy [100]. They are associated with a higher risk of severe hypoglycemia, compared with metformin and more recent glucose-lowering therapies [101], especially in patients with renal or liver disease. Due to hepatic metabolism and renal excretion, sulfonylureas are classically contraindicated in patients with chronic liver or renal disease, despite pharmacokinetics data are very limited in cirrhotic patients [102].

Meglitinides (including repaglinide) are rapid-acting insulin secretagogues that lower postprandial glucose excursions by targeting early-phase insulin release. Unlike sulfonylureas, they have a distinct binding site at the β-cell membrane, resulting in greater insulinotropic effects and a more rapid onset of action [103]. Glinides are characterized by shorter half-life compared to sulfonylureas and they do not have significant renal excretion. Despite the fact that they are metabolized in the liver, there are no large-scale studies that have assessed the efficacy and safety of repaglinide in T2DM patients with chronic liver disease [103,104]. However, there is no obvious information supporting a greater risk of severe hepatotoxicity in diabetic patients with mild liver disturbances [105].

## 9. Insulin

Insulin therapy is the treatment of choice for T2DM associated with advanced liver disease, such as Child-Pugh class C cirrhosis, and can be used in all patients with cirrhosis regardless of the severity of liver impairment, unlike the other antdiabetic drugs. However, several studies and a meta-analysis have been showed that insulin therapy promotes the increase of body weight in T2DM [106]. For this reason, delay in initiation or intensification of insulin therapy is frequent, especially in obese patients [107], body weight reducing drugs (such as GLP-1 analogues or SGLT-2 inhibitors) or drugs that don’t modify body weight (such as metformin or DPP-4 inhibitors) are generally preferred and considered safer than insulin and other body weight-increasing antidiabetic drugs (such as glitazones). However, recent real-world data suggest that gain in body weight is significantly lower in obese than in normal- and overweight patients [108].

Furthermore, insulin is a growth-hormone with well-known oncogenic effects that are carried out mainly through the increase of cell proliferation and the production of insulin-like growth factor-1 (IGF-1) [109]. Insulin levels are increased in diabetic patients treated with exogenous insulin and this has been showed to be associated with an increased risk of colorectal cancer in patients with T2DM [110]. Although a meta-analysis showed that association between insulin treatment and increased risk of colorectal cancer is not significant [111]. Hyperinsulinemia is commonly observed in T2DM, obesity and then in NAFLD and represents a biologic mechanism underlying the association between T2DM and several types of solid neoplasms, including HCC [112]. Furthermore, the association between T2DM and risk of HCC is independent of cirrhosis or chronic liver disease [113]. Case-control and longitudinal studies suggested that insulin may increase risk of HCC in patients with cirrhosis [34,114], while others did not confirm this harmful effect. A meta-analysis of seven observational studies showed that insulin use was associated with a significant increased risk of HCC, compared to non-use (OR 2.61, 95% CI 1.46–4.65) and this oncogenic effect was confirmed regardless of study design and the concomitant effect of other antidiabetic drugs. For unclear reasons, risk of HCC in patients treated with insulin was higher in Asian population. The potential risk of cancer progression in patients with increased insulin concentrations is still controversial. In fact insulin could have a pro-oncogenic effect because it favors cell proliferation and IGF-1 production. On the other side insulin is used principally in obese patients with uncontrolled glycemic levels. These two factors can be also both implicated in cancer progression. Furthermore, no RCTs are available about this topic. Because of these reasons it should be underlined that hyperglycemia should be more harmful as promoting oncogenesis, and the overall benefits resulting from glucose-lowering effects may exceed the potential risks, even with insulin. More studies should be performed to solve this dispute.

For these reasons, insulin can not be considered the ideal drug to treat T2DM in NAFLD patients and its use should be reserved to patients with advanced cirrhosis who could not receive other antidiabetic drugs or patients in which T2DM is poorly controlled with oral antidiabetics.

## 10. Antidiabetic Drugs Use in Liver Cirrhosis

T2DM is a common comorbidity in liver cirrhosis, with a prevalence of 37% [115], and the use of antidiabetic drugs in patients with cirrhosis is debated, because adverse events may be more common and treatment targets may be different from diabetic patients without cirrhosis. Otherwise, the presence of T2DM in liver cirrhosis increases the risk of liver and not liver-related complications and death, so pharmacological approaches to this clinical setting should be safe and should improve survival.

- Metformin. In the past, many clinicians were worried about prescribing metformin in diabetic patients with cirrhosis for the risk of lactic acidosis and liver injury and sometimes metformin were discontinued after diagnosis of cirrhosis. However, a large cohort study showed that the continuation of metformin use after cirrhosis diagnosis significantly improved survival in all stages of cirrhosis, suggesting that metformin is safe and well tolerated also in patients with decompensated liver disease [116]. Particularly, it was shown that metformin had a protective effect only in patients with NASH-related cirrhosis, probably due to the pleiotropic effects of metformin in cell proliferation and differentiation, in apoptosis and inflammation and in metabolic pathways of glucose and lipid homeostasis [117]. However, it should be highlighted that this study was retrospective and did not evaluated competing risk associated with other antidiabetic drugs. Although no RCT was designed to confirm the efficacy of metformin in improvement of survival of diabetic patients with cirrhosis, to date metformin is considered safe and well tolerated in patients with cirrhosis. However, it should be not used in patients with Child-Pugh class C cirrhosis and in presence of severe renal impairment for the risk of lactic acidosis.
- Glitazones, incretines, DPP-4 inhibitors and SGLT-2 inhibitors. These classes of antidiabetic drugs should not be used in Child-Pugh class C cirrhosis, while their use in patients with compensated cirrhosis is safe and could have a positive impact on liver-related outcomes, as previously showed. In patients with Child-Pugh class C cirrhosis, insulin therapy remains the treatment of choice for co-existing T2DM.

## 11. Conclusions

Non-alcoholic fatty liver disease represents one of the most frequent conditions in the world and the rate of patients affected is rapidly increasing. One of the risk factors mostly correlated with this condition is diabetes. For this reason a high number of patients with NAFLD presents at the same time diabetes, in particular type 2 diabetes mellitus, and in this setting of patients there is a higher proportion of inflammation and hepatic damage. Many drugs have been tested to improve hepatic steatosis or to avoid the progression to cirrhosis, with contrasting results. Between these ones, many trials on antidiabetic drugs, like metformin, DPP-4 inhibitors, SGLT-2 inhibitors or thiazolidinediones, were published. Most of the drugs showed an improvement in weight and in liver enzymes, but data regarding histology are lacking and with no accordance.

Antidiabetic drugs seems to be promising drugs, because they are able to treat both NAFLD manifestations and diabetes, preventing worsening of hepatic damage. Because of these reasons and because of the absence of other specific therapies for patients with NAFLD or NASH, it is fundamental to better clarify hepatic safety of antidiabetics. All antidiabetic drugs can be used in patients with compensated cirrhosis, but there are still no data about patients with advanced cirrhosis, especially for glitazones, incretines, DPP-4 inhibitors and SGLT-2 inhibitors.

## Figures and Tables

**Table 1:** Studies assessing the effect of antidiabetic drugs on metabolic and liver outcomes in patients with nonalcoholic fatty liver disease.

Antidiabetic Class	First Name Author, Year of Publication	Trial Design	Patients	Age	Male (%)	BMI	Diabetes	Therapy Dosage and Duration	Body Weight	HOMA-Index	Liver Enzymes	Histological Steatosis	Lobular Inflammation	Hepatocellular Ballooning	Fibrosis
DPP-4 inhibitors	*Cui, 2016*	Sitagliptin vs. Placebo	25	52.9	52	31.9	48	100 mg/die, 24 weeks	Not Improved	Not Improved	Not Improved	Not Assessed	Not Assessed	Not Assessed	Not Assessed
	*Joi, 2017*	Sitagliptin vs. Placebo	6	56.7	50	35.9	100	100 mg/die, 24 weeks	Not Improved	Not Improved	Not Improved	Not Improved	Not Improved	Not Improved	Not Improved
	*Sayari, 2018*	Sitagliptin vs. Sitagliptin + Placebo	138	42.9	60	29.6	NA	50 mg/die, 16 weeks	Improved	Not Assessed	Improved	Not Assessed	Not Assessed	Not Assessed	Not Assessed
	*Hussain, 2016*	Vildagliptin vs. Placebo	29	28	62	30.7	NA	100 mg/die, 12 weeks	Improved	Not Assessed	Improved	Not Assessed	Not Assessed	Not Assessed	Not Assessed
Metformin	*Uygun, 2004*	Metformin plus diet versus diet alone in NASH	18	41	62	29.2	0	1.5 g, 6 months	Improved	Improved	Improved	Not Assessed	Not Improved	Not Assessed	Not Improved
	*Bugianesi, 2005*	Metformin versus vit. E versus diet in NAFLD	55	42	73	28.7	0	2 g, 12 months	Improved	Improved	Improved	Improved *	Improved *	Not Assessed	Improved *
	*Idilman, 2008*	Metformin versus Rosiglitazone vs. diet and exercise alone. 20 NASH	74	47	59	31.5	NA	1.7 g, 12 months	Improved	Improved	Improved	Improved	Improved	Not Assessed	Not Improved
	*Haukeland, 2009*	Metformin versus Placebo in NASH	20	47	73	30.8	20	2.5–3 g, 6 months	Improved	Not Improved	Improved	Improved	Not Improved	Not Improved	Unchanged
	*Shields, 2009*	Metformin plus diet versus diet alone in NASH	19	47	68	32.6	0	0.5–1 g, 12 months	Improved	Improved	Improved	Not Improved	Not Improved	Not Improved	Not Improved
	*Nar, 2009*	Metformin plus diet versus diet alone in NAFLD	34	47	26	31	100	1.7 g, 6 months	Improved	Improved	Improved	Not Assessed	Not Assessed	Not Assessed	Not Assessed
	*Omer, 2010*	Metformin versus Rosiglitazione versus Metformin plus Rosiglitazone in NAFLD	44 **	49	59	31.6	70	1.7 g, 12 months	Improved in both	Not Improved	Improved (NS) in Metformin group. Improved in combination group	Worsed (NS) in Metformin group. Improved in combination group	Worsed (NS) in Metformin group. Improved in combination group	Worsed (NS) in Metformin group. Improved in combination group	Worsed (NS) in Metformin group. Improved in combination group
	*Krakoff, 2010*	Metformin versus Placebo in NAFLD	1073	51	34	34	IFG	1.7 g, 36 months	Improved	Not Assessed	Improved	Not Assessed	Not Assessed	Not Assessed	Not Assessed
	*Garinis, 2010*	Metformin plus diet versus diet alone in NAFLD	20	41	10	36.5	0	1 g, 6 months	Improved	Improved	Not Improved	Not Assessed	Not Assessed	Not Assessed	Not Assessed
	*Tock, 2010*	Metformin plus lifestyle change versus lifestyle changes alone in NAFLD	21	17	NA	>30	0	1 g, 12 months	Improved	Improved	Not Improved	Not Assessed	Not Assessed	Not Assessed	Not Assessed
	*Lavine, 2011*	Metformin versus Vit. E versus Placebo	57	13	82.5	34	0	1 g, 24 months	Not Improved	Not Improved	Not Improved	Not Improved	Not Improved	Improved	Not Improved
	*Sofer, 2011*	Metformin versus Placebo in NAFLD	32	52	53	32.6	19	1.7 g, 4 months	Not Assessed	Not Improved	Not Improved	Not Assessed	Not Assessed	Not Assessed	Not Assessed
	*Hajiaghamohammadi, 2012*	Metformin versus Pioglitazone versus Silymarin	22	33	64	27	0	500 mg, 2 months	Improved	Improved	Improved	Not Assessed	Not Assessed	Not Assessed	Not Assessed
	*Shavakhi, 2013*	Metformin plus probiotics versus Metformin plus Placebo	70	40	46	NA	0	1 g, 6 months	Improved	Not Assessed	Improved	Not Assessed	Not Assessed	Not Assessed	Not Assessed
	*Feng, 2017*	Metformin versus Liraglutide versus Gliclazide	31	46	65.5	27	100	2 g, 6 months	Improved	Not Assessed	Improved	Not Assessed	Not Assessed	Not Assessed	Not Assessed
Thiazolidinediones	*Sanyal, 2004*	Vit.E vs. Vit.E + Pioglitazone	10	47	60	32.5	no	30 mg/die, 6 months	Not Assessed	Not Assessed	Not Improved	Not Improved	Improved	Improved	Not Improved
	*Belfort, 2006*	Diet + Placebo vs. dieta + Pioglitazone	26	51	53	33		30–45 mg/die, 6 months	Improved	Not Assessed	Improved	Improved	Improved	Improved	Improved
	*Idilman, 2008*	Diet and phisical activity vs. Diet and phisical activity + rosiglitazone	24	47.9	77	31.2		8 mg/die, 12 months	Improved	Improved	Improved	Improved	Improved	Improved	Not Improved
	*Ratziu, 2008*	rosiglitazone vs. Placebo	32	53	59	31.5	28	4–8 mg/die, 12 months	Not Improved	Improved	Improved	Not Improved	Not Improved	Not Improved	Not Improved
	*Aithal, 2008*	lifestyle + Placebo vs. lifestyle + piglitazone	37	52	70	30.5	0	30 mg/die, 12 months	Not Improved	Not Assessed	Improved	Improved	Improved	Improved	Not Improved
	*Sanyal, 2010*	Placebo vs. vit.E vs. Pioglitazone	80		41	34	0	20 mg/die, 24 months	Not Improved	Not Assessed	Improved	Improved	Improved	Not Improved	Not Improved
	*Torres, 2011*	rosiglitazone vs. rosiglitazone + Metformin vs. rosiglitazone + losartan	108	49.7	63	33	16	8 mg/die, 12 months	Improved in pio+met	Improved	Improved	Improved	Improved	Improved	Improved
	*Cusi, 2016*	diet vs. diet + Pioglitazone	50	52	72	34.3	48	45 mg/die, 18 months	Improved	Improved	Not Improved	Improved	Improved	Improved	Improved
	*Tikkainene, 2004*	rosiglitazone vs. Metformin	9	50	30	30.6	100	8 mg/die, 4 months	Not Improved	Not Assessed	Improved	Not Assessed	Not Assessed	Not Assessed	Not Assessed
	*Omer, 2010*	Metformin vs. Rosglitazone vs. Metformin + Rosglitazone	62	48.9	48.4	30.6	100	4 mg/day, 12 months	Improved in Met+Ros	Improved in Ros	Improved	Improved	Not Assessed	Not Assessed	Not Improved
	*Gupta, 2010*	Pioglitazone vs. Pioglitazone + diet vs. Metformin	6	57	45	35	100	30 mg/die, 4 months	Improved in pio+dieta	Not Assessed	Improved	Not Assessed	Not Assessed	Not Assessed	Not Assessed
	*Shah, 2011*	Insulin + Pioglitazone vs. Insulin + Placebo	12	na	na	35	100	45 mg/die, 4 months	Not Improved	Not Assessed	Not Assessed	Not Assessed	Not Assessed	Not Assessed	Not Assessed
	*Hajiaghamohammadi, 2012*	Pioglitazone vs. Metformin vs. silimarin	22	33	63.4	27.36	na	15 mg/die	Not Improved	Improved	Not Improved	Not Assessed	Not Assessed	Not Assessed	Not Assessed
	*Ito, 2017*	Pioglitazone vs. ipragliflozin	34	59	53	29.9	100	15 mg/die, 6 months	Not Improved	Improved	Improved	Not Assessed	Not Assessed	Not Assessed	Not Assessed
	*Yaghoubi, 2017*	lifestyle vs. Pioglitazone vs. fenofibrate	30	35	NA	26	NA	30 mg/die, 12 weeks	Improved	Not Assessed	Improved	Not Assessed	Not Assessed	Not Assessed	Not Assessed
SGLT-2 inhibitors	*Ito, 2017*	ipragliflozin vs. Pioglitazone	32	57.3	44	30.7	100	50 mg/die, 24 weeks	Improved	Not Improved	Improved	Not Assessed	Not Assessed	Not Assessed	Not Assessed
	*Kuchay, 2018*	Empagliflozin plus standard treatment for diabetes vs. only standard treatment	22	50.7	59	30	100	10 mg/die, 20 weeks	Improved	Not Assessed	Improved	Not Assessed	Not Assessed	Not Assessed	Not Assessed
GLP-1 analogues	*Armstrong, 2016*	Liraglutide versus Placebo in NASH	26	50	69	34.2	35	1.8 mg/day for 48 weeks	Improved	Not Improved	Not Improved	Improved	Not Improved	Improved	Improved
	*Smits, 2016*	Liraglutide versus Sitagliptin versus Placebo in NAFLD	17	61	70.6	32.8	100	1.8 mg/day for 12 weeks	Not Improved	Not Assessed	Not Improved	Not Assessed	Not Assessed	Not Assessed	Not Assessed
	*Khoo, 2017*	Liraglutide versus lifestyle changes in NAFLD	24	44	100	32.2	0	3 mg/day for 26 weeks	Improved	Improved	Improved	Not Assessed	Not Assessed	Not Assessed	Not Assessed
	*Feng, 2017*	Liraglutide versus Gliclazide versus Metformin	29	47	72.4	28.1	100	1.8 mg/day for 24 weeks	Improved	Not Assessed	Improved	Not Assessed	Not Assessed	Not Assessed	Not Assessed
	*Fan, 2013*	Exenatide versus Metformin	49	51	57.1	28.2	100	10 microg twice a day for 12 weeks	Improved	Not Improved	Improved	Not Assessed	Not Assessed	Not Assessed	Not Assessed
	*Shao, 2014*	Exenatide plus insulin versus intensive insulin therapy	15	42	50	30.6	100	10 microg twice a day for 12 weeks	Improved	Not Assessed	Improved	Not Assessed	Not Assessed	Not Assessed	Not Assessed

* Second biopsy was available in only 17 patients treated with Metformin and in no cases of control groups; ** 22 only Metformin, 22 Metformin plus rosiglitazone. DPP-4: dipeptidyl peptidase-4, SGLT-2: sodium-glucose cotransporter 2, GLP-1: glucagon-like pepide-1, IFG: impaired fasting glucose

## Abbreviations

NAFLD	non-alcoholic fatty liver disease
NASH	non-alcoholic steatohepatitis
T2DM	type 2 diabetes mellitus
HbA1c	glycosylated haemoglobin
PPAR-γ	peroxisome proliferator-activated receptor-γ
RCT	randomized controlled trial
FPG	fasting plasma glucose
FFA	free fatty acids
IR	insulin resistance
HCC	hepatocellular carcinoma
CVD	cardiovascular disease
ROS	reactive oxygen species
TZD	Thiazolidinediones
GLP-1	Glucagon-like pepide-1
SGLT-2	Sodium-glucose cotransporter 2
DPP-4	Dipeptidyl peptidase-4
